# Supplementary material for: Baroreceptor Sensitivity Predicts Functional Outcome and Complications after Acute Ischemic Stroke
Source: J Clin Med. 2019 Mar 3;8(3):300. doi: 10.3390/jcm8030300 (PMC6462921; doi:10.3390/jcm8030300)
Supplement: Supplementary file 1 [file jcm-08-00300-s001.pdf]

Baroreceptor sensitivity predicts functional outcome and complications after acute ischemic stroke

Ching-Huang Lin, MD[1,2,4], Cheng-Chung Yen, MD[2], Yi-Ting Hsu, MS[1,2], Hsin-Hung Chen, PhD[3], Pei-Wen Cheng, PhD[3], Ching-Jiunn Tseng, MD, PhD[3], Yuk-Keung Lo, MD, PhD[2], Julie Y.H. Chan, PhD[1,5]

[1]Department of Biological Sciences, National Sun Yet-Sen University, Kaohsiung, Taiwan; [2]Section of Neurology, [3]Department of Medical Education and Research, Kaohsiung Veterans General Hospital, Kaohsiung, Taiwan; [4]Department of Physical Therapy, Shu-Zen Junior College of Medicine and Management, Kaohsiung, Taiwan; [5]Institute for Translational Research in Biomedicine, Kaohsiung Chang Gung Memorial Hospital, Kaohsiung, Taiwan

Corresponding author:

Julie Y.H. Chan, PhD

Institute for Translational Research in Biomedicine, Kaohsiung Chang Gung Memorial Hospital, No.123, DAPI Rd. Kaohsiung City, 83301, Taiwan

E-mail: jchan@cgmh.org.tw

Tel: 886-7-7317123, ext. 8576

Supplementary Table 1: Multiple logistic regression of dependency on post-stroke 3 months, 6 months and 12 months

Variables	Dependency-3 months				Dependency- 6 months				Dependency- 12 months			
	OR	*95% CI*		*P*	***OR***	*95% CI*		*P*	***OR***	*95% CI*		*P*
BRS	1.39	0.66	2.92	0.383	1.05	0.47	2.31	0.914	1.85	0.77	4.43	0.166
NIHSS (ER)	1.31	1.17	1.48	<0.001	1.29	1.16	1.43	<0.001	1.29	1.16	1.43	<0.001
Age	1.05	1.02	1.09	0.003	1.03	1.00	1.06	0.094	1.04	1.00	1.08	0.029
HTN	1.17	0.40	3.44	0.781	1.36	0.41	4.49	0.618	0.76	0.22	2.59	0.656
DM	2.13	1.02	4.47	0.045	2.87	1.33	6.19	0.007	2.34	1.03	5.33	0.043
Smoking	1.02	0.50	2.10	0.959	1.23	0.58	2.62	0.597	1.66	0.73	3.75	0.226
IV tPA	0.37	0.13	1.07	0.066	0.51	0.18	1.46	0.212	0.59	0.20	1.74	0.341

Note. OR: Odds ratio, CI: Confidence interval, mRS: modified Rankin scale, BRS: baroreflex sensitivity, NIHSS (ER): NIH stroke scale at emergency room, HTN: Hypertension, DM: Diabetes, IV tPA: Intravenous tissue type plasminogen activator.
